# Engaging adolescents in changing behaviour (EACH-B): a study protocol for a cluster randomised controlled trial to improve dietary quality and physical activity

**DOI:** 10.1186/s13063-020-04761-w

**Published:** 2020-10-15

**Authors:** Sofia Strömmer, Millie Barrett, Kathryn Woods-Townsend, Janis Baird, David Farrell, Joanne Lord, Leanne Morrison, Sarah Shaw, Christina Vogel, Wendy Lawrence, Donna Lovelock, Lisa Bagust, Judit Varkonyi-Sepp, Patsy Coakley, Lyall Campbell, Ross Anderson, Tina Horsfall, Neelam Kalita, Olu Onyimadu, John Clarke, Cyrus Cooper, Debbie Chase, Danielle Lambrick, Paul Little, Mark Hanson, Keith Godfrey, Hazel Inskip, Mary Barker

**Affiliations:** 1 MRC Lifecourse Epidemiology Unit, Southampton General Hospital, University of Southampton, Southampton, UK; 2 NIHR Southampton Biomedical Research Centre, University Hospital Southampton, NHS Foundation Trust, Southampton, UK; 3 Southampton Education School, Faculty of Social Sciences, University of Southampton, Southampton, UK; 4 School of Computing, Engineering and Built Environment, Glasgow Caledonian University, Glasgow, UK; 5 Southampton Health Technology Assessments Centre, University of Southampton, Southampton, UK; 6 Centre for Clinical and Community Applications of Health Psychology, University of Southampton, Southampton, UK; 7 Hampshire County Council, Winchester, UK; 8 Southampton City Council, Southampton, UK; 9 School of Health Sciences, Faculty of Environmental and Life Sciences, University of Southampton, Southampton, UK; 10 School of Primary Care, Population Sciences and Medical Education, University of Southampton, Southampton, UK; 11 Human Development and Health Academic Unit, Faculty of Medicine, University of Southampton, Southampton, UK

**Keywords:** Adolescence, Behaviour change, Body composition, Cluster randomised controlled trial, Cost-effectiveness, Diet, Digital intervention, Education, Physical activity

## Abstract

**Background:**

Poor diet and lack of physical activity are strongly linked to non-communicable disease risk, but modifying them is challenging. There is increasing recognition that adolescence is an important time to intervene; habits formed during this period tend to last, and physical and psychological changes during adolescence make it an important time to help individuals form healthier habits. Improving adolescents’ health behaviours is important not only for their own health now and in adulthood, but also for the health of any future children. Building on LifeLab—an existing, purpose-built educational facility at the University of Southampton—we have developed a multi-component intervention for secondary school students called Engaging Adolescents in Changing Behaviour (EACH-B) that aims to motivate and support adolescents to eat better and be more physically active.

**Methods:**

A cluster randomised controlled trial is being conducted to evaluate the effectiveness of the EACH-B intervention. The primary outcomes of the intervention are self-reported dietary quality and objectively measured physical activity (PA) levels, both assessed at baseline and at 12-month follow-up. The EACH-B intervention consists of three linked elements: professional development for teachers including training in communication skills to support health behaviour change; the LifeLab educational module comprising in-school teaching of nine science lessons linked to the English National Curriculum and a practical day visit to the LifeLab facility; and a personalised digital intervention that involves social support and game features that promote eating better and being more active.

Both the taught module and the LifeLab day are designed with a focus on the science behind the messages about positive health behaviours, such as diet and PA, for the adolescents now, in adulthood and their future offspring, with the aim of promoting personal plans for change. The EACH-B research trial aims to recruit approximately 2300 secondary school students aged 12–13 years from 50 schools (the clusters) from Hampshire and neighbouring counties. Participating schools will be randomised to either the control or intervention arm. The intervention will be run during two academic years, with continual recruitment of schools throughout the school year until the sample size is reached. The schools allocated to the control arm will receive normal schooling but will be offered the intervention after data collection for the trial is complete. An economic model will be developed to assess the cost-effectiveness of the EACH-B intervention compared with usual schooling.

**Discussion:**

Adolescents’ health needs are often ignored and they can be difficult to engage in behaviour change. Building a cheap, sustainable way of engaging them in making healthier choices will benefit their long-term health and that of their future children.

**Trial registration:**

ISRCTN 74109264. Registered on 30 August 2019. EACH-B is a cluster randomised controlled trial, funded by the National Institute for Health Research (RP-PG-0216-20004).

## Background

### Background and rationale

The NHS Long Term Plan sets out a prevention agenda in the UK aimed at reducing the risk of developing non-communicable diseases (NCDs) such as cardiovascular conditions and type 2 diabetes [1]. Insufficient exercise and poor dietary quality are common and are linked to increased risk of NCDs. NCDs place a heavy burden on society, hospitals and community health services, costing the NHS £7 billion a year [2].

UK adolescents have poorer diets than other age groups, and fewer than 20% meet physical activity guidelines [3, 4]. Intervening during adolescence to support better health habits can bring a triple benefit: to the immediate health and wellbeing of the young person, to their own health in adulthood and to the health of the next generation [5–9]. It is well-established that improving the dietary quality and nutritional status of both young women and young men before conception improves pregnancy and birth outcomes and therefore the long-term health of the offspring [10–12]. It has also been suggested that adolescence is a critical period during which optimal nutrition could mitigate the effects of poor fetal and infant nutrition [13, 14].

As a critical period of both physical and social development, adolescence is the time during which the physiological, mental and behavioural foundations of long-term health are consolidated. Peak muscle and bone mass as well as cardio-respiratory fitness are reached during adolescence, and these physiological processes are both nutritionally sensitive and predictive of later health [15–17]. In addition, widespread brain re-modelling during adolescence leads to a large increase in cognitive ability [18]. Adolescence is also a key time for the development of executive function and the capacity to make independent choices, follow them through and achieve goals, as well as the ability to form healthy social networks. Lifelong behaviour patterns are established in adolescence, including choices about diet and physical activity (PA) [13].

Adolescence is a challenging time to intervene to improve health behaviours for both psychological and physiological reasons. Adolescents find it difficult to engage with the long-term consequences of their lifestyle choices. Developmental changes in brain structure leave them sensitive to emotional and social influences and to prioritising the immediate over the long-term; brain pathways involved in decision-making processes do not mature fully until early adulthood [14, 19]. Systematic reviews suggest that motivated and engaged adolescents can improve their health behaviours [20]. However, little is known about precisely how to motivate and engage adolescents in sustaining positive changes long term [21, 22].

The latest research evidence strongly indicates that successful interventions with adolescents are as follows: (i) multi-component, (ii) involve schools, (iii) engage and motivate adolescents to change their health behaviours and (iv) involve social support from friends and parents [23, 24]. In addition, digital platforms show potential as complementary features in complex interventions targeting health behaviour change and are particularly relevant to this age group. Approximately 83% of 12–15 year olds owned smartphones in 2018, and 99% spent an average of 20 h a week online [25]. Key strategies for effective engagement with digital interventions are recognised to include co-designing interventions with adolescents, the personalisation of interventions and connectivity to peers and the user’s wider social networks [26]. It is increasingly recognised that interventions need to facilitate collaboration between different agencies such as schools, community and parent groups and not rely on one setting, such as the school or family.

Interventions to improve adolescents’ diet and PA have been implemented with varying success; effective engagement with, and motivation of, adolescents remains a pertinent issue. Gender-specific issues should not be overlooked, and positive effects post-intervention may not be apparent in the short-term, making medium and longer-term measures important [24, 27, 28]. Many interventions favour combining health and nutrition education with behavioural skills training, even though evidence suggests that adolescents are not ignorant about the health implications of their food choices and PA habits, nor are they motivated by health in the distant future [29, 30]. Recent research has suggested that interventions designed to support adolescent health may be more engaging and successful if they align health agendas with adolescents’ own values and priorities [30, 31]. EACH-B is designed using a person-based approach [32–34] with extensive co-creation to maximise alignment with adolescents’ own values in order to make the intervention both engaging and effective. The views and input of parents and adolescents in the age range of EACH-B’s target population have been continually sought and incorporated into the trial design through extensive engagement work with local schools, youth groups, and through LifeLab’s Young Ambassadors scheme.^1^ In addition, two advisory groups have been set up to ensure the intervention design and delivery are acceptable to parents of young teenagers and to the young people themselves. A number of ‘Game Jams’ involving approximately 300 adolescents have been run throughout the development phase, in order to ensure the app reflects the values and priorities of the intended user group [35].

### Hypothesis

Aligning an intervention design with adolescent values and using fun, engaging methods of delivering behaviour change support as part of a multi-component, school-based intervention improves diet and PA habits of secondary school students.

## Methods

### Aim/objectives

The aim of this cluster randomised controlled trial is to evaluate whether EACH-B, a complex intervention designed to engage, motivate and support adolescents aged 12–13 years, improves their dietary quality and PA habits.

### Trial design

EACH-B is a cluster randomised controlled trial using a 1:1 allocation within a superiority framework. The intervention consists of three-components: (1) face-to-face support from teachers trained in skills to support behaviour change, (2) engagement in the LifeLab school-based education programme and (3) a digital intervention with games as well as peer- and parent-support features.

### Participants and study setting

We propose to evaluate EACH-B through a cluster randomised controlled trial. We plan to recruit boys and girls of middle academic ability in Year 8 (aged 12–13 years) from 50 state secondary schools/academies (approximately 2300 students) to take part in the trial. Year 8 is the second year of senior school in the UK and was chosen to take part in EACH-B in consultation with schools for two key reasons: schools are better able to deliver the intervention at this time, before students start their GCSE curriculums in Year 9 and timetabling becomes more difficult; adolescents in Year 8 often have increased levels of independence in terms of food choices whilst travelling to and from school alone or with friends. Schools in Hampshire, UK, and the surrounding counties will be eligible to take part. Hampshire is a large county (pop. 1.4 million) in the south of England with a wide range of socioeconomic profiles. Some rural areas of Hampshire are affluent, but the two major cities Southampton and Portsmouth are in the most deprived quintile of local authorities in the UK [36]. Schools will be recruited from both rural and urban settings in order to reflect the diversity of the population (see the ‘Randomisation/blinding’ section for more on randomisation procedures).

Each school will be randomly allocated to either ‘control’ or ‘intervention’ status. Of the schools recruited, 25 will therefore be intervention schools where two classes of Year 8 students will complete the LifeLab module, be offered support from teachers trained in skills to support health behaviour change and receive the digital intervention. The other 25 schools will form the control group and will receive normal schooling.

### Eligibility criteria

All state secondary schools and academies in Hampshire and surrounding areas are eligible to take part in the EACH-B intervention trial. Independent and selective schools including special schools and single-gender schools are excluded from taking part because by nature of being selective their inclusion could bias the study findings.

### The intervention

In late 2019, a successful pilot trial was run with 170 students from six schools in the Southampton area, to test and modify the intervention. The intervention comprises:

i) Professional development for teachers including training in communication skills to support health behaviour change, known as ‘Healthy Conversation Skills’ (HCS), explained in detail below
ii) LifeLab educational module comprising in-school teaching of nine science lessons linked to the English National Curriculum and a hands-on practical day visit to LifeLab, held part way through the module
iii) A personalised digital intervention (the ‘app’) with social support and game features

#### Professional development for teachers

The EACH-B intervention includes professional development (PD) for all teachers involved in delivering the LifeLab educational module. The 1-day PD training course takes place at LifeLab and focuses on science education relevant to the implementation of the nine lessons in school. It offers access to online support materials which describe the underpinning science. Teachers are trained in HCS [37, 38] to engage with their students in making plans to improve their diet and/or activity levels via a personal ‘LifeLab pledge’. Teachers are trained how to support their students to keep their pledges, and how to use the digital intervention, through asking open questions and listening rather than telling. An additional HCS training session will be offered to the whole staff body in intervention schools to enhance the opportunity for adolescents to be supported at school to improve their health behaviour.

HCS training was developed in Southampton to provide communication skills to support behaviour change. These skills were designed in the first instance for health and social care practitioners to use with their patients and clients, but have since been adapted to the training of teachers to enable them to better support behaviour change in their students. While health promotion is not seen as a core requirement of a teacher’s role, we have seen high levels of engagement from teachers throughout the development work and EACH-B pilot trial. It is widely acknowledged both in the scientific literature and by schools that diet and PA behaviours are significant factors in both academic performance and student wellbeing and there is growing evidence that health and health behaviours have measurable consequences for attainment [39]. Being more physically active at age 11 is associated with higher attainment at GCSE, while being obese at age 11 is associated with lower attainment [40, 41]. Children from more disadvantaged backgrounds are more likely to be overweight/obese, have poorer diets and be less physically active. Being overweight/obese can reduce children’s self-esteem, which may lead to lower educational attainment and behavioural problems [39]. Therefore, schools and teachers are keen to learn skills that enable them to support students to eat well and be more active.

The use of HCS encourages people to reflect on behaviours that they would like to change, in many cases making the unconscious, habitual behaviours conscious and therefore amenable to deliberate change. HCS trains people to use five key skills: (1) creating opportunities for having healthy conversations; (2) asking open ‘discovery’ questions that lead people to explore and find their own solutions; (3) listening more than talking and so empowering people to identify and take control of their own behaviour change; (4) reflecting on practice in order to be more effective; and (5) supporting goal-setting using SMARTER^2^ action planning, providing people with a sense of change and progress. These skills were originally developed in collaboration with local health service commissioners in Southampton, whose needs-assessment found that their healthcare providers lacked confidence to support clients to improve their diets and lifestyles [42]. HCS training recognises that skills to support behaviour change need to go beyond education and instead empower individuals to take control of their health behaviours and to problem-solve. As with Motivational Interviewing, the training offers an approach to supporting behaviour change that is based on the understanding that giving people information is insufficient to change their behaviour; they must also be motivated to change and have the tools to implement that change.

HCS training is philosophically underpinned by Bandura’s social cognitive theory of the socio-environmental and personal determinants of health [43]. Self-efficacy is a central construct in this theory and describes an individual’s belief that he or she is capable of carrying out a specific behaviour, which implies that he or she also has the knowledge and skills to do so. HCS are designed to increase self-efficacy through empowering problem-solving, and employ Behaviour Change Techniques [44, 45] intended to support small changes in behaviour, leading to acquisition of mastery skills which Bandura proposes as a means of raising self-efficacy. Training in HCS is designed to increase the self-efficacy and hence build the capacity of practitioners and clients and, in doing so, change the ethos of those practitioners and their organisations to one that empowers change. The EACH-B intervention is designed to operate both at the level of individual behaviour change and at the level of changing the culture of schools to trigger automatic as well as reflective processes underlying behaviour change [37, 38, 46, 47].

#### LifeLab educational module

The LifeLab educational module aims to engage adolescents with the knowledge and understanding needed to enable them to make appropriate health choices—their health literacy—and to motivate them to change their dietary and PA behaviours. The theme of the module is ‘Me, My Health & My Children’s Health’, and it is delivered in an interactive and highly engaging format which sets scientific knowledge into a relevant and accessible context for this age group [4]. The educational module is designed to be delivered as four pre- and five post-LifeLab visit lessons delivered in science classes during the school day.

The materials used in the educational module are explicitly linked to the English National Curriculum, embed the messages of the LifeLab visit and have been updated specifically for the EACH-B trial. For example, an additional lesson focusing on the influences of the food environment on healthy lifestyle choices has been added, in order to encourage the adolescents to critically analyse their own food environments and the influence these may have on their dietary behaviours.

Health messages in the module are linked to both the hands-on practical activities the students will carry-out on the day visit to LifeLab and to the school-based activities. This approach is intended to ensure that the adolescents understand the long-term implications of their current diet and PA on their future health, their children’s health and on the risk of NCDs for both.

#### Practical day visit to LifeLab facility

Halfway through the LifeLab educational module in school, the students and their science teacher have a day visit to the purpose-built laboratory facility, based in Southampton General Hospital. The visit combines a mixture of hands-on practical work, reflection on lifestyle choices and learning about the science behind health messages. Activities include:

- Experiencing a variety of ways to measure health including assessing carotid artery blood flow and structure using ultrasound, measuring body composition, performing lung function tests, training in CPR and testing grip strength and flexibility
- Extracting their own DNA and carrying out gel electrophoresis experiments that illustrate how a healthy diet can induce epigenetic changes that alter DNA structure and are passed from parents to offspring, with implications for cardiovascular and lifelong health for themselves and their children
- Small group discussion sessions with scientists based at the hospital, to introduce students to the range of career options in scientific disciplines.

At the end of the LifeLab visit, and with support from LifeLab staff, students are encouraged to make a ‘pledge’ about a positive change for their own health. Students also download the EACH-B app onto their personal devices during the day (see below).

#### Personalised digital intervention

The digital intervention will be in the form of a mobile phone application (app) with game features. It has been developed using a person-based approach to intervention development, combined with user-centred design principles for digital game design and a participatory design process. The design of the game is underpinned by self-determination theory and employs a range of behaviour change techniques [33, 44, 45].

During the LifeLab visit, students will be asked to download the app onto their personal mobile devices (Android or IOS) and log in. Any student without a personal device will be given instructions for downloading the app at home via a shared family device. The app will involve creating a character and choosing games, quizzes and challenges to complete. Players can choose challenges and none are compulsory. The app will allow players to connect with each other if they wish. Parents/carers of students in the intervention will also be offered a companion app to help them support their adolescent in making healthy lifestyle choices. The parent app includes information about the different elements of the app developed for the young people taking part in the intervention. It also contains ideas, suggestions and prompts as to how parent and adolescent can join forces to improve food choices and activity levels for the whole family.

### Outcomes

All outcomes will be measured twice, once at baseline and again 12 months later at follow-up.

#### Primary outcomes

The trial has co-primary outcomes for dietary quality and PA. Dietary quality will be assessed by a 20-item food frequency questionnaire (FFQ). This FFQ has been developed specifically for use with adolescents using data for boys and girls aged 11–18 years who took part in the National Diet and Nutrition Survey. Principal component analysis was applied to these data in order to identify 20 indicator foods which best describe better and poorer dietary quality of UK adolescents. This FFQ has shown good comparison with important nutritional biomarkers including 25-hydroxy vitamin D, total carotenoids, serum folate and vitamin C. Using GENEActiv™ accelerometers PA will be assessed as minutes of daily low, moderate and vigorous physical activity (LMVPA), also described as total PA [48]. At baseline and again at the 12-month follow-up, GENEActivs will be worn for 7 days and the output data will be averaged over this period, or the maximum period of valid data.

#### Secondary outcomes

Secondary outcomes for dietary quality are as follows: usual portions in the past month of water, sugar sweetened beverages (SSBs), chips and crisps and usual portions of fruit and vegetables consumed in a typical day. The number of portions of fruit and vegetables are analysed separately to estimate daily fruit and vegetable consumption for each adolescent [49]. Categories of PA will also be assessed as secondary outcomes, namely average acceleration, intensity gradient, sedentary time, light PA, light to moderate PA, moderate to vigorous PA, LMVPA 1-min, LMPA 1-min and MVPA 1-min. The categories of ‘1-min’ restricts to activity that has a minimum of 1-min bout duration. All PA outcomes (primary and secondary) will be analysed separately for activity at the following times: weekdays, weekends, during school hours and during out-of-school hours.

Additional secondary outcomes are as follows: BMI *z*-scores, with and without adjustment for pubertal status as indicated by standing height, sitting height and weight [50]; self-reported frequency of PA from a modified version of the Youth Physical Activity Questionnaire (YPAQ) validated for use in 12–13 year olds [51]; behavioural self-regulation and self-efficacy for healthy eating and PA; and quality of life and wellbeing measured by two age-appropriate tools: the Child Health Utility 9D (CHU9D) [52] and the Cantril Ladder [53]. Behavioural regulation and self-efficacy for PA will be assessed by the Behavioural Regulation for Exercise Questionnaire [54] and the PA section of the Self-Efficacy for Healthy Eating and Physical Activity measure (SE-HEPA) [55]. Behavioural regulation and self-efficacy for diet will be assessed using the recently developed Confidence and Behavioural Autonomy (CBA) scale. This is age-specific and has been validated against the healthy eating scales of the SE-HEPA and the Treatment Self-Regulation Questionnaire (TSRQ) [56].

### Participant timeline

**Fig. 1:**
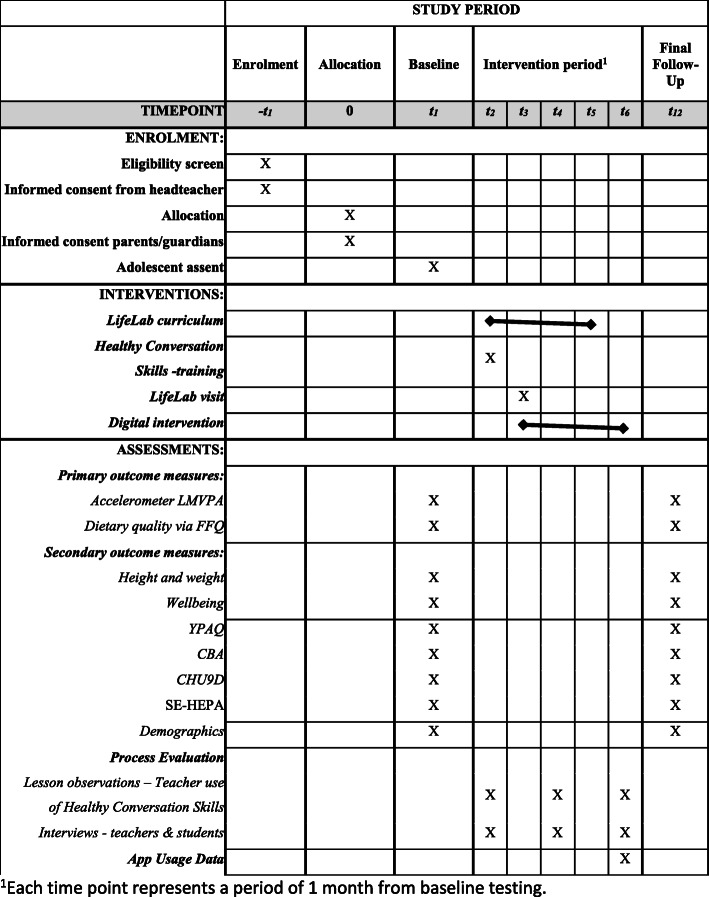
EACH-B schedule of enrolment, interventions and assessments. Superscript digit one indicates each time point represents a period of 1 month from baseline testing

A schematic schedule of enrolment, interventions and assessments is shown in Fig. 1.

### Sample size

Unpublished analysis of earlier data from LifeLab indicates an intra-school (class) correlation coefficient of 0.035. Forty-six schools each sending two classes amounting to approximately 50 students from each school and 2300 students in total will provide 90% power at a 0.025% significance level (accounting for two primary outcomes) to detect a 0.25 SD difference in diet quality score or minutes of total PA in intervention and control schools. Comparable effect sizes have been considered in other health interventions as meaningful in terms of change in health behaviours, and our level of 0.25 SDs falls in the mid-range of effect sizes reported in a meta-synthesis of meta-analyses of behaviour change interventions in the general population [57]. We will recruit two Year 8 classes (12–13 years) from 50 schools allowing for drop-out, though in previous LifeLab studies only one school has ever dropped out. To minimise bias from loss of students to follow-up, we will request class lists for each participating class, so that missing participants at each stage of the follow-up can be identified and included in secondary analyses and process evaluation assessing uptake of the intervention.

### Recruitment

Recruitment for EACH-B began in September 2019 and will run for 2 years, with data collection taking place at schools at baseline and again 12 months later. The LifeLab team has worked with schools for many years and good systems for recruiting schools have been developed, so recruitment difficulties are not anticipated. We appreciate that control schools will not want to miss out on the intervention and therefore all control schools will be offered a visit to LifeLab the following year. The schedule of enrolment for the trial is shown in Fig. 1.

Schools will be recruited through a range of methods including presentations at relevant local meetings such as the Secondary Heads of Science forum meetings, and letters sent to Head Teachers and Heads of Science of eligible schools in the recruitment catchment area. The recruitment pack for EACH-B includes a cover letter and information sheet for the school, offering basic information about the trial and explaining how the experience will differ for control and intervention schools. Schools are then offered a meeting with the EACH-B research team at which further details are discussed and any questions answered. This meeting will take place with the Head of Science and a member of the Senior Leadership Team at the school, at a time to suit them. It is also an opportunity for the research team and the school staff to establish how the intervention will run in the school, if it is allocated to the intervention arm, as each school operates differently in terms of timetabling science classes.

Schools will be asked to allocate two middle ability Year 8 classes to participate in the trial totalling approximately 50 students. The teaching programme is designed for students in this age group and of all ability levels; there are no exclusion criteria for students. For students who may require more input (those who have English as a second language, for example), we provide support for schools in planning delivery of the module. Specifics are discussed at the teachers’ PD day. Schools will already have in place provision for these students, and so it is a matter of ensuring that the LifeLab materials are accessible by all participating students.

Following the meeting at school, the Head Teacher is asked to sign a consent form confirming they wish to take part, that they understand the trial procedures and to name the two classes that will participate.

### Randomisation/blinding

After signed agreement from the schools has been obtained and the classes taking part have been identified, schools will be randomised to receive usual schooling (control) or the EACH-B intervention (LifeLab programme, HCS training for teachers, and access to the digital intervention) (see Fig. 1). We will use a minimisation procedure developed by the Southampton Clinical Trials Unit (TENALEA), which aims to achieve a balance of schools in the two arms based on the following three criteria:

- The proportion of students in the school receiving free school meals (cut-off > 24%);
- The proportion of students in the school achieving L5 GCSE (equivalent to a high ‘C’ grade) in English and Maths (cut-off > 40%);
- Whether or not the school already participated in the full LifeLab programme in the previous 2 years.

The randomisation is administered through a web-based secure system to which the EACH-B team submit the details of schools who have consented to participate. These are sequentially numbered and the allocation to intervention or control is then reported to the investigators. Blinding from this point onwards is not possible except of the statisticians who will be analysing the data.

Many schools we recruit will have previously been involved in LifeLab. Contamination of the intervention effect is unlikely to occur as the Year 8 students taking part in EACH-B will not have visited LifeLab before, and except for siblings, they will generally have limited contact with older children in the school. It will not be possible to blind schools and their students to their allocation due to the nature of the intervention.

### Data collection, management and analysis

#### Data collection methods

Primary and secondary outcome data will be collected at baseline and follow-up visits by research staff to schools, conducted during class time. Standing and sitting height and weight will be collected by trained research nurses from the Clinical Research Facility at the Southampton NIHR Biomedical Research Centre (SBRC). These measures will be used to derive body mass index *z*-scores and biological maturity [49]. All researchers and research nurses working on the EACH-B trial will be trained in trial-specific procedures and be required to complete appropriate safeguarding and eating-disorder training.

Questionnaire data will be collected through participant completion of questionnaires on iPads during the baseline and follow-up visits to schools. The class will be divided into small groups of 7–8 students working with one member of the research team who will act as a facilitator. Before students begin completing the questionnaires, the facilitator will use a trial-specific standard operating procedure to explain key points about the questionnaires and will remain with the group throughout the session to answer any questions that might arise.

GENEActiv™ accelerometers will be distributed to participants by trained research staff during the data collection sessions at both time points. The devices will be programmed to automatically start measuring at midnight on the first day of data collection and stop measuring precisely 7 days later, in order to capture both weekend and weekday activity. A sampling frequency of 100 Hz will be used. Participants will be asked to keep the device on their non-dominant wrist for seven full days, preferably without taking it off at any point. Seven days after the baseline data collection visit, schools will be asked to return the GENEActivs to the research team via courier or another secure method.

#### Data management

After data collection visits to schools, all data will be downloaded from the iPads via a Secure Sockets Layer (SSL) that encrypts the data before sending it and storing it in the database. The database itself is kept on a University server. All questionnaire data will be kept in accordance with General Data Protection Regulations (GDPR), University of Southampton Data protection policy and in accordance with the protocols of the MRC Lifecourse Epidemiology Unit LEU). The data will be stored in password-protected computers by the research team and only accessible by them. Data will be stored in Access databases and managed with support from the data management staff of the MRC LEU, who have extensive data management expertise and manage data from more than 200 studies. After the trial is complete, anonymised data will be available to other researchers under our data sharing protocols.

Identifying information will be collected about participants, purely for the purposes of matching baseline and follow-up questionnaires. All identifying information will be stripped from the rest of the data after linkage is complete and will be stored separately. It will only be kept in case a further follow-up is planned.

#### Statistical methods

Data will be analysed using Stata, SPSS and Mplus. The primary analyses will be according to the intention-to-treat principle, comparing dietary quality and PA levels in the intervention and control groups using mixed effects linear regression to account for clustering within schools. The main analysis will compare these outcomes at baseline and at 12 months follow-up. Although randomised at the level of schools using a minimisation algorithm, there may still be disparities between the intervention and control participants at baseline. These will be assessed prior to analysis and relevant confounders will be incorporated in the models; factors to be considered include gender, exact age at recruitment and household area of deprivation using the Income of Deprivation Affecting Children Index (IDACI) score [58]. Adjustment for baseline dietary quality and PA levels will be included in the relevant models to allow an assessment of change from baseline. Sensitivity analyses will examine effects of missing data, and multiple imputation will be used where appropriate, accounting for the clustered nature of the data.

Comparisons for secondary outcomes will also be modelled using mixed effects linear regression, with the use of binary models for binary outcome variables. Mixed effect logistic regression will be used for rare outcomes and mixed effect binary regression (or Poisson regression with robust variance if the binary regression models fail to converge) will be used for outcomes that occur in more than 10% of students. We will then conduct a mediation analysis to examine the role of these secondary factors in mediating the effect of the intervention on primary outcomes. The main analyses will be conducted using the latest available version of Stata.

Planned subgroup analyses will focus on whether there are different effects for boys and girls, differing ethnicities, seasonal variation, IDACI score and estimated biological maturity in outcomes. We will also determine the effect of EACH-B on outcomes for those who fully engage with the digital intervention (per protocol analysis) and assess uptake of the digital intervention by gender, ethnicity and IDACI score. Complier average causal effect (CACE) modelling techniques will be used to examine factors that predict engagement with intervention components and to examine intervention effects specifically for students who engaged with those components. As this is a cluster randomised controlled trial, many of the assumptions underlying this method are unlikely to be valid, most obviously the independence of the participants [59]. These assumptions will be assessed and methods of analysis developed appropriately. The CACE analysis will be performed using MPLus.

### Data monitoring

Data monitoring will be the responsibility of the trial team at the MRC LEU. The Steering Committee will receive regular data reports as part of their bi-annual meetings. Due to the low risk nature of the trial, a separate Data Monitoring Committee is not deemed necessary.

#### Harms/auditing

We are not expecting the trial to give rise to any adverse events or harms. However, a risk assessment was completed and submitted as part of the ethical approval process. All staff involved in visiting schools have the enhanced level of DBS to work with children, are trained in safeguarding and awareness of eating disorders and have basic levels of awareness about dealing with someone who may become anxious for any reason during a data collection visit.

### Process evaluation

Using the MRC guidance on process evaluation of complex interventions [60], focusing on the programme logic model, we will use mixed-methods to examine (i) implementation, (ii) context and (iii) mechanisms of impact of the EACH-B intervention.

i) *Implementation*: we will examine how intervention delivery is achieved and what is delivered (fidelity, dose, adaptations and reach) by, for example, monitoring downloads of the digital intervention on LifeLab day as a proportion of those eligible, frequency of access to the digital intervention, as well as conducting structured, qualitative observations of teacher/student interactions, and teacher/student interviews.
ii) *Context*: we will assess context at school level by interviewing relevant staff about other activities and factors that may affect how the intervention was implemented and how it worked. The wider policy context will also be assessed at local and national levels by considering relevant healthy living initiatives or campaigns and their potential influence.
iii) *Mechanisms of impact*: we will conduct interviews with students, teachers and parents to explore their experiences of and engagement with the intervention as a whole and use in-app telemetry to explore usage of the digital intervention. Some interviews will be carried-out with specific subgroups of students to ensure that the intervention is not stigmatising.

#### Qualitative process evaluation

Analysis of the qualitative data collected as part of the process evaluation will be conducted with a view to achieving a comprehensive understanding of the way in which an intervention like EACH-B is implemented and how that relates to the outcomes. Thematic analyses of all qualitative data from the process evaluation will be undertaken. Structured, qualitative observations of teacher/student interactions and interviews with students, teachers and parents to explore their experiences of and engagement with the intervention will be analysed using various forms of content analysis. For observations of teacher/student interactions, structured record forms will be designed to monitor use of HCS. Interviews will be audio-recorded, transcribed verbatim and analysed using inductive thematic analysis and a standard methodology [61]. Initial codes will be discussed between coders to reach agreement on themes, and then discussed with the wider research team and PPI panels. Broad themes will then be broken down to identify commonly expressed themes and unusual cases. Approximately 10% of the data will be coded by two team members to check that the coding scheme is identifying all the themes and concepts and that there is a shared understanding of what they are. Findings will be used to assist with interpretation of the trial outcomes and to illuminate mechanisms through which the intervention has its effect.

#### Economic evaluation

A model will be developed to estimate the cost-effectiveness of EACH-B compared to usual schooling. The model will extrapolate short-term observed effects on diet, PA and quality of life (CHU9D) to estimate future health impacts and societal costs. There are many risks associated with poor diet and low PA over the life course, including increased incidence of a range of NCDs and adverse social, economic and well-being outcomes. We will focus on four key risks for which there is good evidence of a short to medium-term impact: incidence of type 2 diabetes, mental health, low birth weight and future loss of earnings [62]. Health outcomes will be quantified using quality-adjusted life years (QALYs), including direct effects of diet and PA on quality of life (CHU9D), as well as losses associated with type 2 diabetes, depression and low-birth weight pregnancies. Costs will be estimated from a societal perspective, including costs to schools, local authorities and the NHS and loss of earnings for individuals. Costs and QALYs will be estimated over a time horizon of 20 years in the base case, discounted at UK recommended annual rates [63]. A range of sensitivity and scenario analyses will be conducted to assess uncertainty of the model predictions. This will include alternative assumptions about the persistence of observed effects on diet quality, PA and quality of life from the trial.

## Discussion

This trial will estimate the effectiveness of a complex intervention to improve diet and PA in adolescents, designed to have reach and affordability. If it proves effective, the intervention could be rapidly and inexpensively disseminated to all secondary school students attending LifeLab from across the Wessex region. Potential for the intervention to be introduced widely across the UK will be explored. Some elements of the intervention will be easier to translate than others. In areas where there is already an educational intervention providing initial engagement for adolescents in thinking about their health, educators could be trained in communication skills to support behaviour change. The supplementary digital intervention is low-cost and sustainable. If successful in supporting behaviour change, the intervention has potential for both immediate impact on adolescents’ health and well-being and for improving the health of the nation for generations to come.

The intervention is designed to deliver outcomes aligned to the local authority’s Sustainability and Transformation Plan. As such, it represents an attempt to meet the need to provide preventive methods that can easily be up-scaled and that deliver technological solutions for major health issues. Trial findings that will have wide application and impact include improvements in understanding how best to intervene with maximal effectiveness and cost-effectiveness to improve adolescent health behaviours, and to engage, and sustain the engagement, of adolescents. Sub-group analyses of data will allow tailoring of the intervention to specific groups, e.g. the most disadvantaged, hardest to reach, or boys as distinct from girls. The programme provides information about the value of, and best practice in, co-creation of initiatives with adolescents and our understanding of mechanisms of creating change with adolescents.

### Trial status

Recruitment for the RCT initiated in September 2019 and was due to be completed by June 2021. The trial was halted in March 2020 due to the closure of schools in response to the COVID-19 global pandemic. The trial team plans to restart the trial in late Autumn 2020. The trial Protocol is version 1 date 21 August 2019.

## Data Availability

Data sharing is not applicable to this article as it is a protocol of an ongoing study and no data is reported. All anonymised datasets from the study will be deposited in a publicly available repository after the trial has ended.

## Abbreviations

BMI: Body mass index
CPR: Cardio-pulmonary resuscitation
EACH-B: Engaging adolescents in changing behaviour
GDPR: General data protection regulation
HCS: Healthy conversation skills
IDACI: Income Deprivation Affecting Children Index
ISRCTN: International Standard Registered Clinical/soCial sTudy Number
LMVPA: Low, moderate, vigorous physical activity
MRC LEU: Medical Research Council Lifecourse Epidemiology Unit
NCD: Non-communicable disease
PPI: Patient public involvement
RCT: Randomised control trial

## Footnotes

1 LifeLab’s Young Ambassadors Scheme is a scheme whereby young people who visit LifeLab with their school can sign up to be a Young Ambassador in order to support LifeLab’s aims and objectives by taking part in special activities including being consulted on new ideas as they are developed and worked up by the LifeLab team.

2 Specific, Measurable, Action oriented, Realistic, Timed, Evaluated and Reviewed
